# Effect of heat shield locations on rework-induced thermal management in ball grid array solder joint

**DOI:** 10.1038/s41598-022-19436-6

**Published:** 2022-09-06

**Authors:** Adlil Aizat Ismail, Maria Abu Bakar, Abang Annuar Ehsan, Azman Jalar, John Burke, Zol Effendi Zolkefli, Erwan Basiron

**Affiliations:** 1 Western Digital®, Sandisk Storage Malaysia Sdn. Bhd., Plot 301A, Persiaran Cassia Selatan 1, 14100 Seberang Perai Selatan, Pulau Pinang Malaysia; 2 Institute of Microengineering and Nanoelectronics, Universiti Kebangsaan Malaysia, 43600 Bangi, Malaysia; 3 Department of Applied Physics, Faculty of Science and Technology, Universiti Kebangsaan Malaysia, 43600, Bangi Malaysia

**Keywords:** Characterization and analytical techniques, Design, synthesis and processing, Thermodynamics

## Abstract

This study investigated the effectiveness of heat shield placement locations during the rework process to avoid thermal and mechanical damage to adjacent ball grid array components and their solder joints on double-sided printed circuit board assembly. Three types of heat shield placement locations were used: sample X, individual heat shield placement on adjacent components of the rework location; sample Y, a U-shaped, and sample Z, a square-shaped heat shield placed respectively at the heat source location. The dye and pull test results, infrared thermography, and temperature measurements were analysed to understand the relationship between the location of the heat shield and solder joint damage during rework. Heat shield placement at the heat source location on the reworked component can reduce the peak temperatures on the adjacent rework component locations by up to 8.18%. The peak temperatures of the centre and corner of the BGA component can be maintained below 195 °C and 210 °C, respectively to improve the adjacent rework component locations' solder joint quality by reducing solder joint damage by more than 50% solder cracks. This is useful for thermal management during rework involving high-density ball grid array component placements on double-sided printed circuit board assembly.

## Introduction

Printed circuit board assembly (PCBA) rework is frequently used in the manufacturing industry as a beneficial effort to reduce waste and as a result, to boost total company revenue. PCBA rework is becoming increasingly crucial in times of difficulty in obtaining components, increased demand for flexibility, and short product development cycles for the product to be ready for the market^1,2^. The main advantage of reworking a PCBA is that depending on the extent of damage, it can be performed faster than replacing it^3^.

The process of reworking ball grid array (BGA) components is known as an area array rework. The solder joints are concealed beneath the component body making the rework of area-array devices more challenging^4^. The combination of the higher operating temperature requirements of lead-free soldering and the sensitive nature of area-array components makes defining a rework procedure for lead-free BGA components difficult^5^. In high-density product design, multiple BGA components are placed close to each other; hence, the adjacent rework component locations have a high risk of being exposed to thermal reflows during rework^6^. Several obstacles can only be overcome by introducing new or revised methods, such as tighter thermal profiles and extreme precision during PCBA rework procedures^7^.

A heat shield is used to avoid thermal or mechanical damage to the component, printed circuit board (PCB), adjacent rework component locations, and solder joints. The heat shield can minimize the temperature delta between the bottom and top sides of the PCBA during the rework hot air reflow process for BGA removal and assembly, thereby reducing the exposure of heat transfer to adjacent components^8^. Component damage and solder joint cracks can be caused by unintentional reflow of adjacent component solder joints^9^. Due to the interaction between tin-based solder and copper pads, intermetallic compound (IMC) will occur during the assembly process and in service of the solder joints^10^. Low mechanical characteristics of solder may be caused by a very thick IMC layer. Additionally, the IMC's form has a big impact on how reliable solder joints are^11.^ Because of their intrinsic brittleness, thick IMC is easily broken, and longitudinal transform-induced stress caused by minus volume reaction accumulating at the solder/IMC interface and within the IMC layer can cause mechanical property degradation^12^. The heat shield during the rework process also prevents the IMC layers on solder joints of the adjacent components from growing too thick, which could impact the solder joint quality and reliability^13^. Limited studies have addressed thermal management using heat shield during rework involving high-density component placement on double-sided PCBA^14,15^.

The main objective of this study is to investigate the effectiveness of the heat shield placement locations during the rework process to avoid thermal and mechanical damage to the adjacent components of the rework location and their solder joints on both the top and bottom PCBA sides. For this purpose, (a) an infrared thermography camera was used to obtain the thermal distribution on the surface of the BGA components during the rework process; (b) the thermal distribution on the solder joint array was validated using temperature measurements via thermocouple (TC) wires; (c) dye and pull tests were used to determine the post-rework solder joint cracks; and (d) the dye and pull test results and temperature measurements were analyzed qualitatively and quantitatively to further understand the correlation between the heat shield placement locations and the damage to the adjacent components’ solder joints during rework.

## Results and discussion

**Figure 1:**
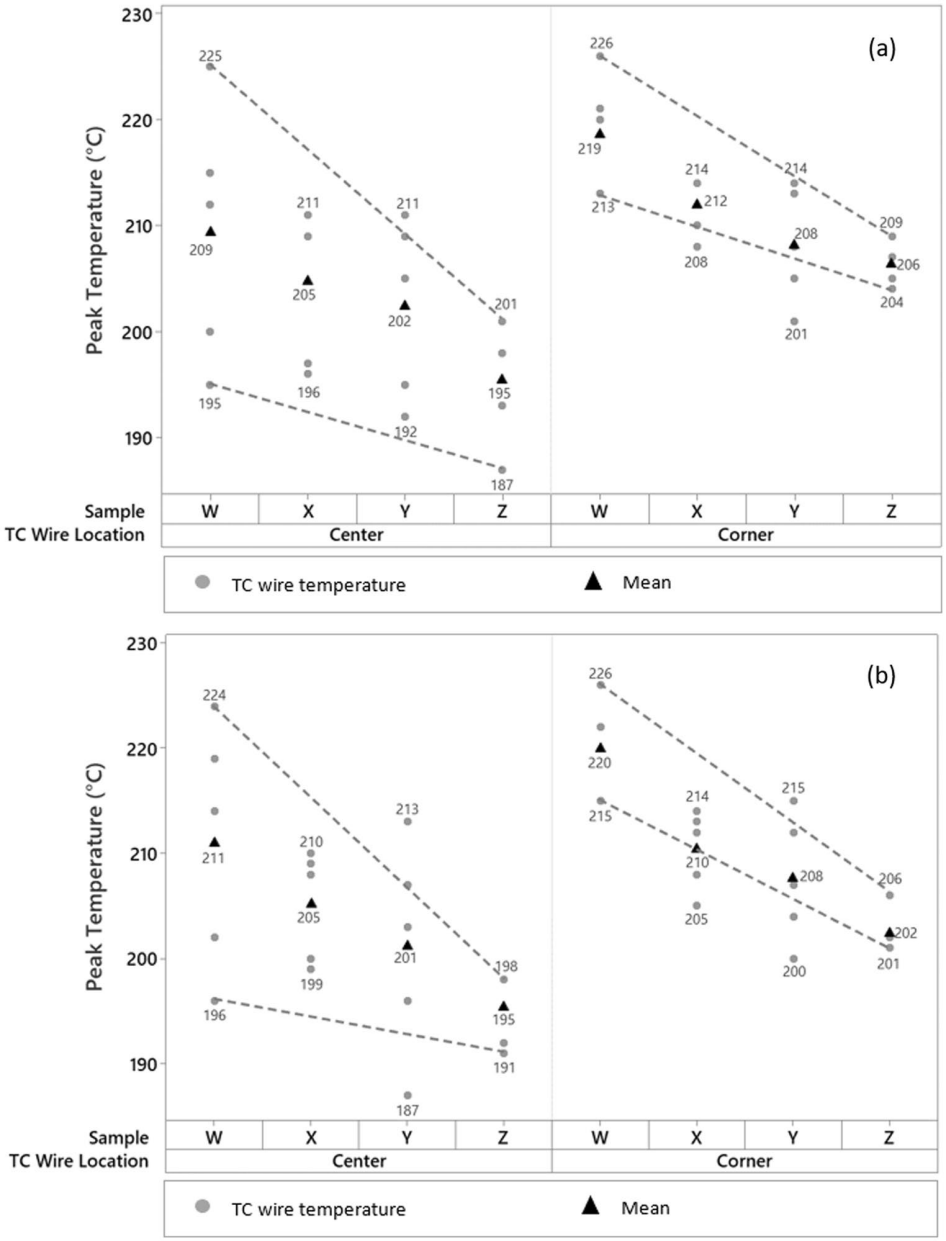
Variability chart for peak temperature on centre and corner of BGA for all samples: (**a**)—top PCBA side; (**b**)—bottom PCBA side.

The peak temperature results for all samples are shown in Fig. 1a, for the surrounding components located on the top PCBA side. Fig. 1b shows the peak temperature results for the mirror component locations located on the bottom side of the PCBA. The variability charts indicate that there was an interaction between the type of heat shield placement locations and the peak temperature for both the centre and corner of the BGA for the top and bottom PCBA sides during the rework process. The highest peak temperature was detected for sample W, which was reworked without a heat shield. For samples X, Y, and Z, reworking by applying heat shield(s) caused the centre and corner temperatures on surrounding components to be significantly lower than sample W.

The range of corner peak temperature for both top and bottom PCBA sides in all samples was smaller than the range of centre peak temperature. This observation agrees with Sommerer et al.^16^, where the position of the TC wires from the heat source location was correlated with the amount of heat absorption. Weng and Martin^17^ also reported that TC temperature readings varied depending on the heat shield locations from the heat source. Samples X and Z had less variation in the centre and corner peak temperatures for both the top and bottom PCBA sides, but sample Z had a lower peak temperature range. Sample Y had a lower minimum peak temperature than samples X and Z, but the variability in the peak temperature range in sample Y was higher with the highest peak temperature, making it ineffective in controlling heat dissipation. The mean for the peak temperatures indicated by the triangular shapes showed a downward trend, indicating a temperature reduction by using the heat shield during the rework process.

**Figure 2:**
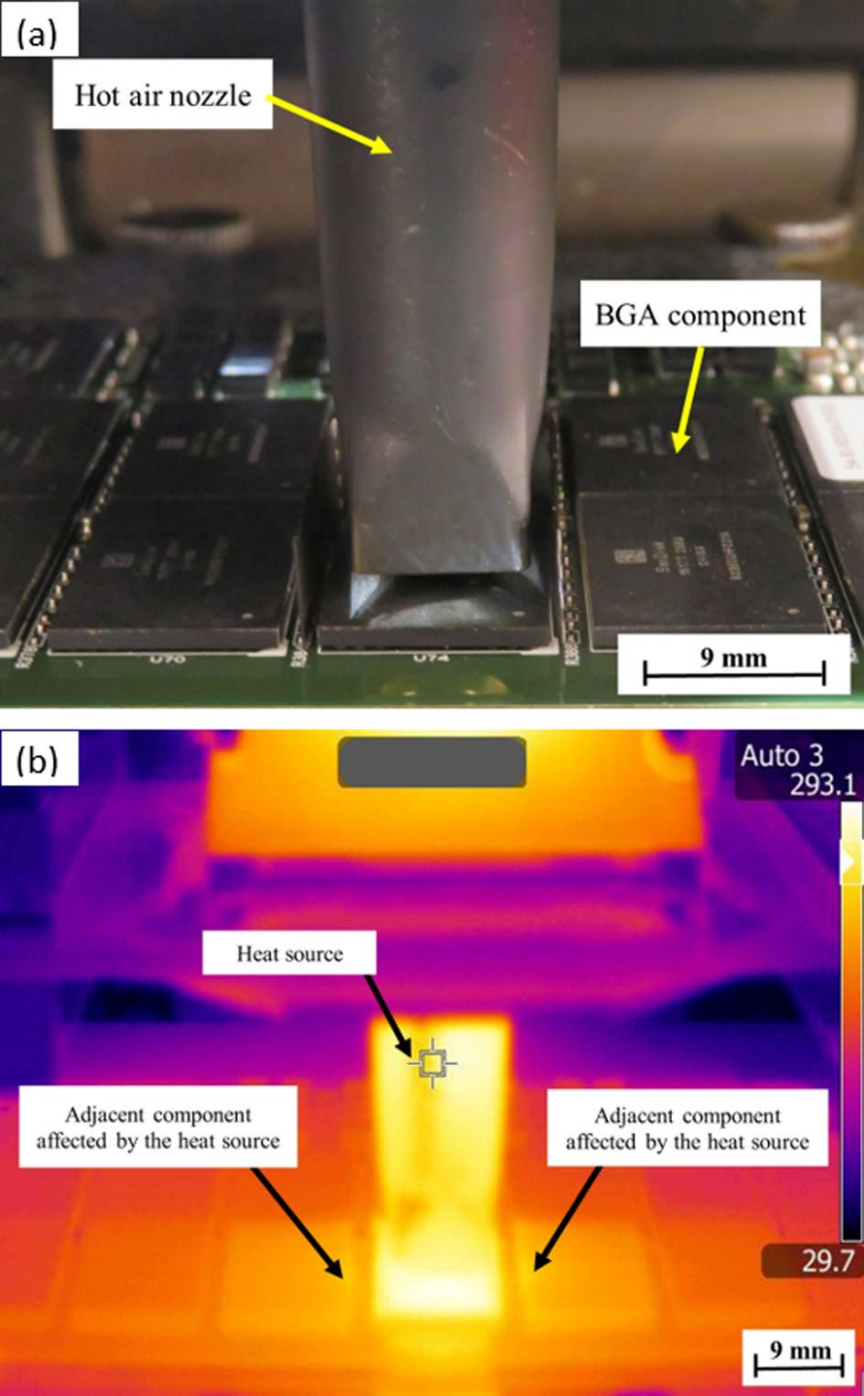
Photograph (**a**) and infrared thermography image (**b**) during the rework process for sample W (no heat shield).

**Figure 3:**
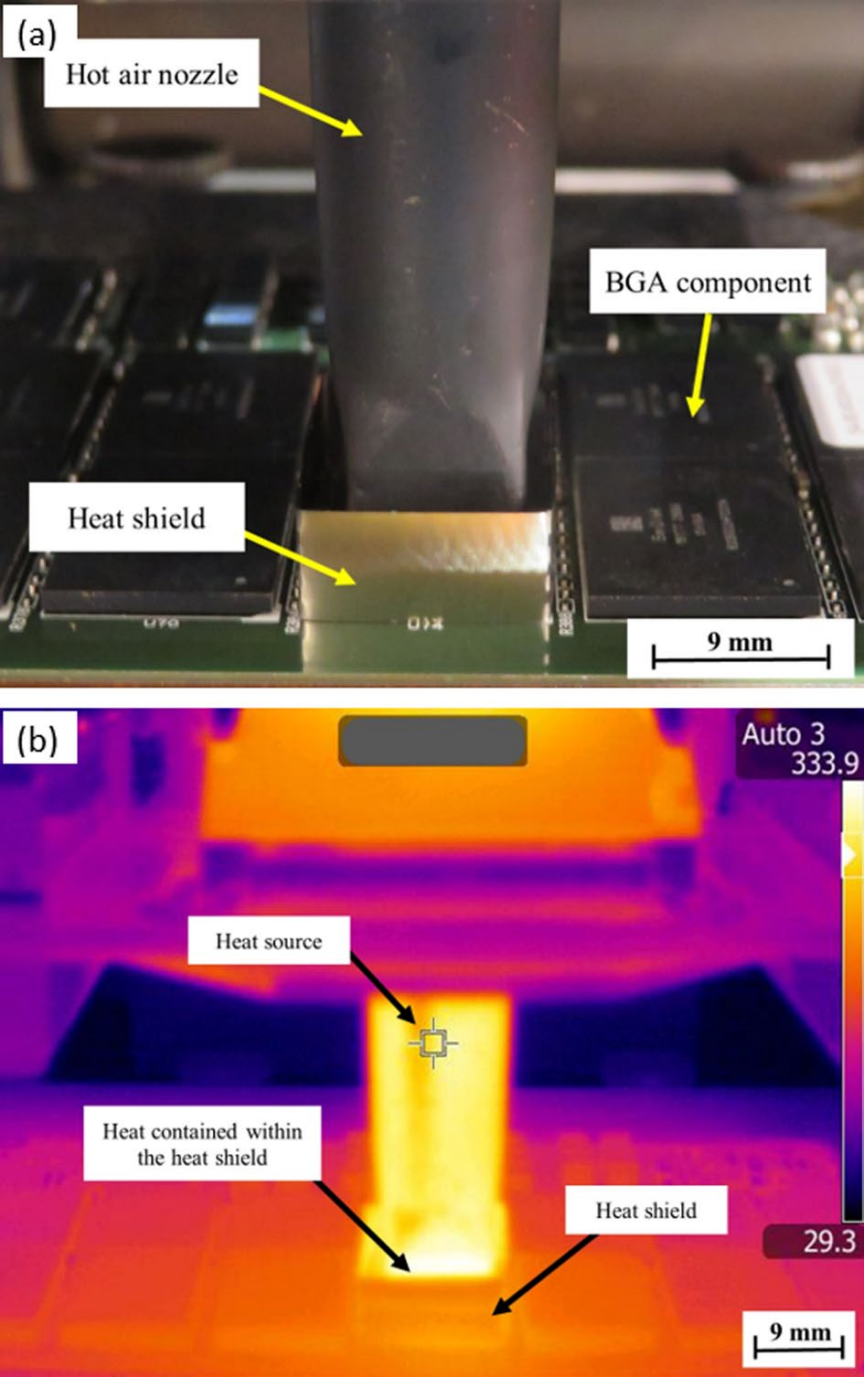
Photograph (**a**) and infrared thermography image (**b**) during the rework process for sample Z (with a square-shaped heat shield.

Validation by infrared thermography confirmed the heat dissipation of the components in the PCBA samples during rework^18^. Warmer temperatures, where more heat and infrared radiation are emitted, are indicated by brighter colours (red, orange, and yellow), while cooler temperatures are indicated by purple and dark blue or black where less heat and infrared radiation are emitted^19^. Here below show pictures of the temperature distribution without heat shield application and Fig. 2 and the heat shield application in Fig. 3. Photographs of the BGA components on the top PCBA side during the rework process were taken with a regular camera as a reference for the infrared thermography images.

The bright yellow colour was the same as the heat source which came from the hot air nozzle can be seen on the side of the adjacent components of the rework location for sample W in Fig. 2b, indicating a surface temperature of 293.1 °C. This was due to the rapid temperature increase along the side of the adjacent BGA components' surface during the rework process. The bright yellow colour from the heat source indicated the surface temperature of 333.9 °C according to the heat scale was properly contained in sample Z, as shown in Fig. 3b. The adjacent components of the rework location were dark orange indicating that the temperature was lower than the heat source. With the heat shield application during the rework process, the temperature of the adjacent BGA components of the rework location decreased, as did the active heat-spreading area.

**Figure 4:**
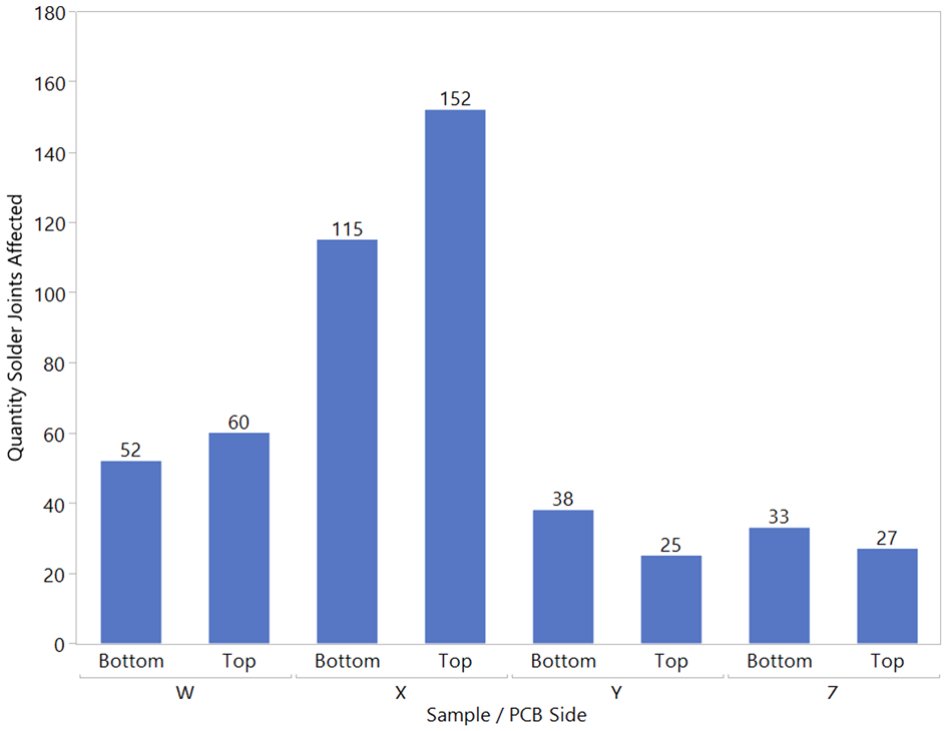
Bar chart on quantity solder joints affected with dye penetration across all samples.

Figure 4 shows the number of BGA solder joints affected by dye penetration for both top and bottom PBCA sides in all samples. The number of solder joints affected in samples Y and Z was reduced from sample W, which showed that the heat shields enabled the reduction of thermal damage on the solder joints. Sample X, using an individual heat shield located on each adjacent component of the rework location, has the highest number of solder joints affected by the dye penetration, despite the temperature of the adjacent components of the rework location being much lower than sample W. This result has deviated from the expected outcome of the heat shield placement location.

**Figure 5:**
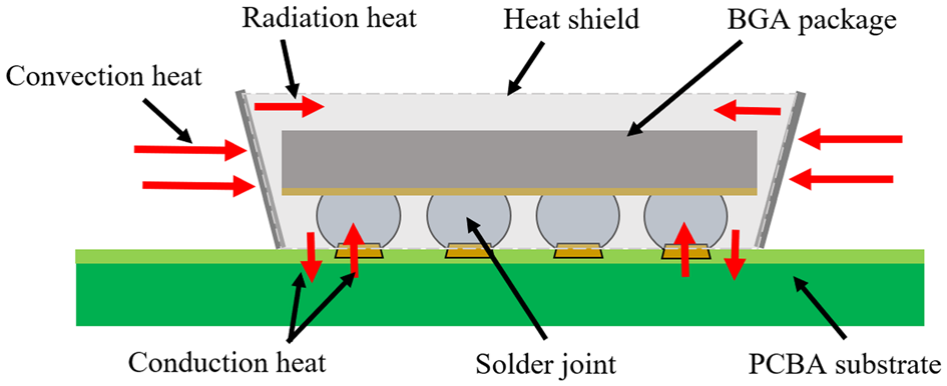
Schematic of the combination of convection, radiation, and conduction heat interactions on the BGA component solder joints in sample X during rework.

The inner heat shield wall generates radiation heat from the hot air convection heat interacting with the outside heat shield wall. The interaction created transient heat transfer from the outside to the inside of the heat shield wall thereby transferring conductive heat toward the PCBA surface and BGA solder joints, as shown in Fig. 5. This aligns with the findings of Stein et al.^20^ on the temperature distribution conditions within the heat shield. Kong et al.^21^ reported that thermal fatigue failure of solder joints can occur in a lower temperature variation range.

**Figure 6:**
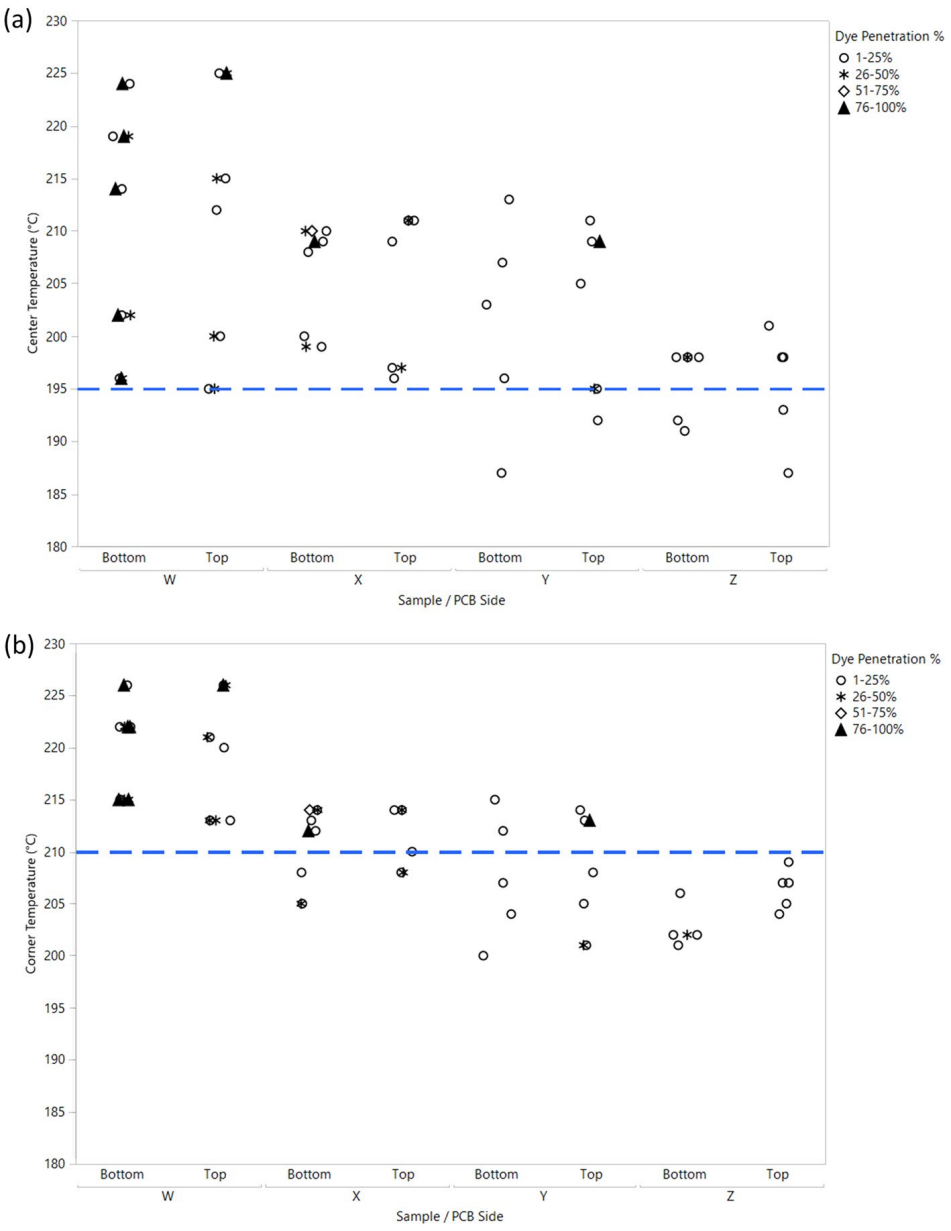
Dye penetration% with the impact of temperature differences at the BGA component area: (**a**)—centre of the BGA components; (**b**)—corner of the BGA components.

The severity of the dye penetration percentage and its correlation with the centre and corner temperatures of each adjacent component of the rework location are shown in Figs. 6a and b, respectively. Sample W had the most severe dye penetration at 76–100% and mainly occurred at the bottom side. This was because no heat shield was applied during rework. Samples X and Y had the same dye penetration percentages at the bottom and top, respectively. In addition, 51–75% dye penetration was observed on the bottom side of sample X. The dye penetration percentage of sample Z was less than 50% on the bottom side. Dye penetration of 51% and above occurred when the centre temperature of the adjacent components of the rework location exceeded 195 °C. Similar results were observed when the corner temperature of the adjacent BGA component exceeded 210 °C.

In this study, the temperature of the adjacent components of the rework location during the rework process can be reduced by addressing the heat shield placement locations. Infrared thermographic images were validated using the TC wires temperature reading to integrate the heat distribution image of the surface temperature of the BGA components and the actual peak temperature of the solder joint array. The sample Z heat shield placement location had the most effective heat reduction on the adjacent components of the rework location, where the mean peak temperatures on the top PCBA side for the BGA component centre and corner were reduced by 6.70% and 6.85%, respectively.

For the bottom PCBA side, the heat reduction for the BGA component's mean peak temperature was 7.58% at the centre and 8.18% at the corner. Dye penetration of more than 50% due to the solder joint crack can be prevented as long as the adjacent components of the rework location temperature can be maintained below 195 °C and 210 °C for the centre and corner of the BGA component, respectively, during the rework process. This finding is in line with Chen et al.^22^, that lowering the temperature exposure to the solder joints will reduce the impact of reliability issues, such as the thickening of the IMC layer that affects the shear strength of the solder joints.

## Conclusion

This study provides a method to address the issue of heat shield placement locations while reworking high-density BGA components placement populated on double-sided PCBA. Thermal management during the rework process was performed effectively using heat shields. Placing the heat shield at the heat source location can reduce the temperature of adjacent components of the rework location during the rework process. This method can be successfully used for reworking high-density BGA components populated on double-sided PCBA. Combining a square-shaped heat shield with placement at the heat source location where the reworked component is located can reduce the peak temperatures on the adjacent components of the rework location by up to 8.18%. It can also maintain a peak temperature below 195 °C and 210 °C for the centre and corner of the BGA component respectively, to improve the solder joint quality by reducing solder joint damage.

## Methods

### Materials and sample description

**Figure 7:**
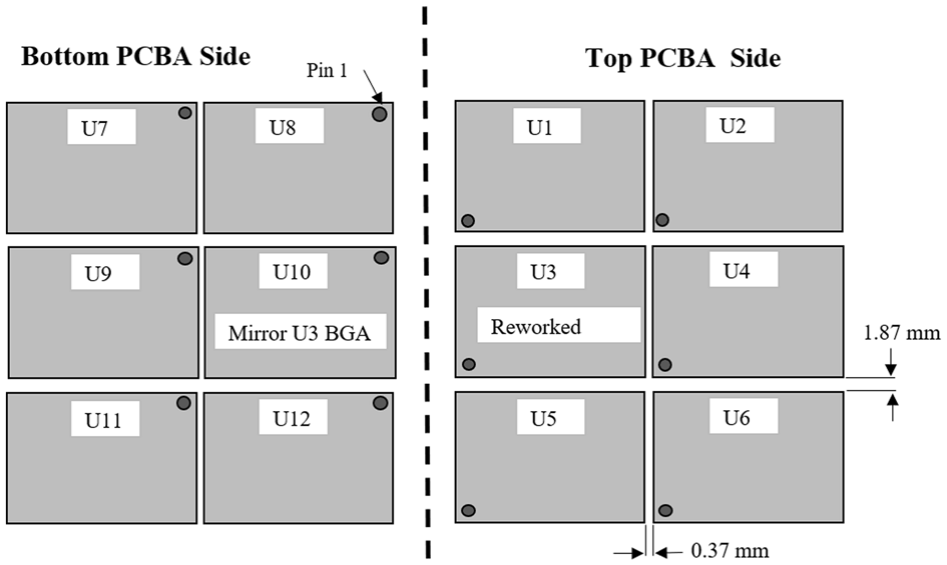
Schematic of BGA components position and reworked component location (U3).

Four variables were used as test vehicles, as shown in Table 1: sample W: rework without a heat shield as a control sample; sample X: rework by placing heat shields on adjacent components of the rework location; sample Y: rework by placing a U-shaped heat shield at the heat source, and sample Z: rework by placing a square-shaped heat shield at the heat source. Twelve BGA components were located at the top and bottom of the PCBA for each sample. There were six BGA components on each side of the PCBA, which mirror each other. For this research, ten adjacent BGA components from the rework area on the top (U1, U2, U4, U5, U6) and bottom (U7, U8, U9, U11, U12) PCBA were studied. The measurement gap between the BGAs is shown in Fig. 7. A BGA component (U3) on the top side of the PCBA was reworked using a rework machine. Alphabet “U” is selected since it is a standard reference designator for an integrated circuit component as per ASME Y14.44-2008^23^.

**Table 1:** Material samples and variables.

Sample	Variable
W	Rework without a heat shield (Control)
X	Rework by placing heat shields at adjacent components of the rework location
Y	Rework by a placing U-shaped heat shield at the heat source
Z	Rework by placing a square-shaped heat shield at the heat source

Each BGA component consists of 132 solder balls with one central column unpopulated. The BGA component solder ball diameter was 0.49 ± 0.5 mm. Both material composition details for the lead-free solder ball and solder paste are listed in Table 2. The solder paste was used for the BGA components assembly before the rework process. The PCB has 14 layers, and the finishing is an organic solderability preservative (OSP) with solder mask-defined lands.

**Table 2:** Solder paste and BGA component solder ball material composition.

Details	Solder paste	Solder joint (ball type)
Alloy composition (wt%)	Sn96.5 Ag3.0 Cu0.5	Sn97.18 Ag2.0 Cu0.75 Ni0.07
Melting temperature (°C)	217	218

The reflow profile was based on a lead-free rework process temperature profile, which required preheating between 100 and 190 °C. The soaking or preheating activation temperature was 140–220 °C for 90 seconds. The component ramp rate was 2–4 °C per second. The reflow dwell temperature was 220–230 °C for 80 seconds. The solder joint peak temperature was maintained at 230 °C for 15 seconds.

### Heat shield material and placement location

**Figure 8:**
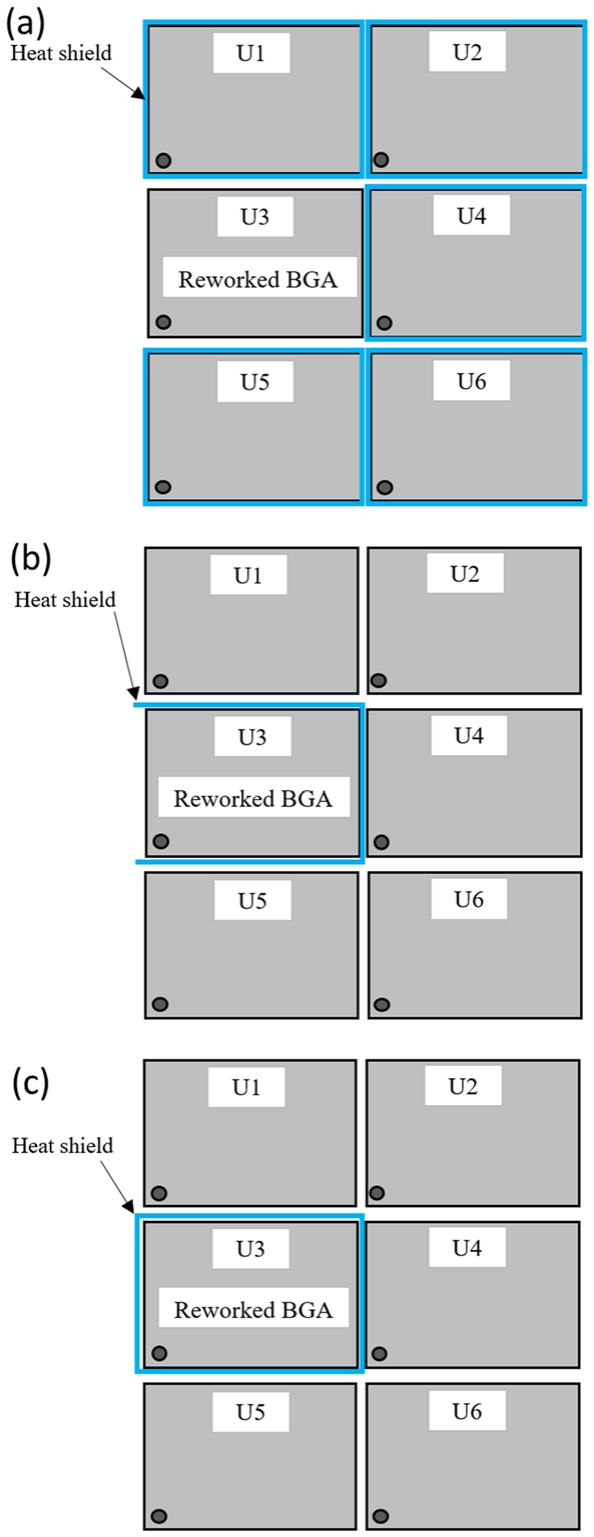
Schematic of heat shield(s) placement locations: (**a**)—an individual heat shield on adjacent components of the rework location; (**b**)—a U-shaped heat shield on the rework component; (**c**)—a square-shaped heat shield on the rework component.

Heat shields were made from stainless steel sheet metal due to their reflectivity, emissivity, thermal conductivity, and specific heat capacity^24^. It is also cost-effective, durable, and adaptable to customization^25^. Three types of heat shield placement locations were used: sample X using individual heat shield placement on adjacent components of the rework location, as shown in Fig. 8a; sample Y using a U-shaped heat shield placement at the heat source location, as shown in Fig. 8b; and sample Z using a square-shaped heat shield placement at the heat source location, as shown in Fig. 8c. The thickness of the stainless steel sheet metal was 0.8 mm. The dimensions of the heat shield were 5 mm in height, 12.69 mm in width, and 18 mm long.

### Rework temperature profiling

**Figure 9:**
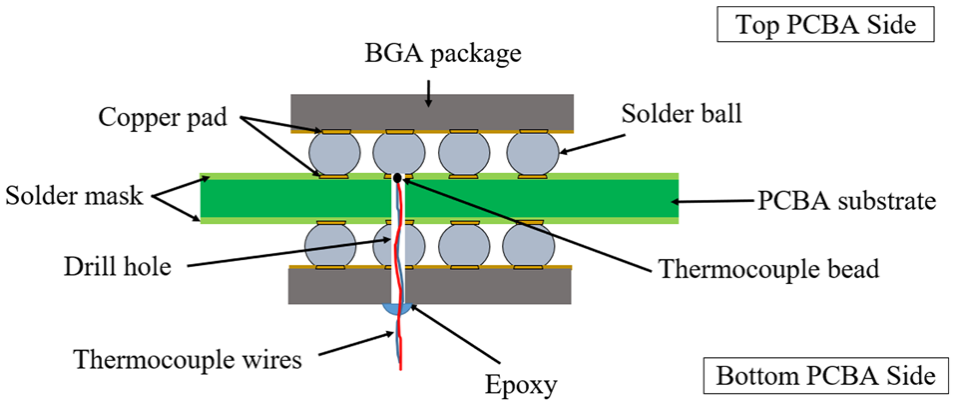
Schematic of TC wire placement on BGA component solder joint.

**Figure 10:**
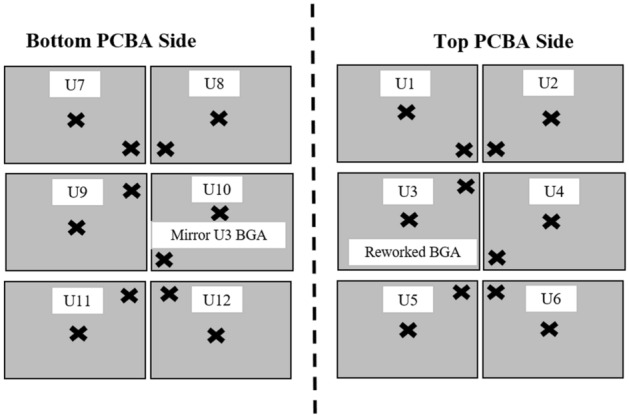
Schematic of TC wires placement locations on reworked and adjacent BGA components of the rework location.

The TC wires were used for rework temperature profiling. The PCBA sample was drilled to properly place the TC bead and wires on the solder joint of the BGA component, which needs to be monitored as per the schematic in Fig. 9. The holes from the drilling process were covered with epoxy resin. The TC wires were placed on the rework, mirror rework, and five adjacent components on both the top and bottom PCBA sides, as shown in Fig. 10. TC wire placements were based on the IPC-7095D-WAM1 recommendations to represent the lowest to highest thermal mass areas^26^. The TC wires were attached to the rework machine to monitor the temperature of the reworked and adjacent components while exposing the targeted rework BGA component location with hot air coming from the hot air nozzle and bottom convection heater. For this experiment, only temperatures of adjacent BGA components of the rework location on the top and bottom PCBA were recorded and analyzed.

### Rework method

The PCBA samples were baked in an oven for 9 hours at 125 °C to remove moisture and prevent thermal shock on the PCBA^27^. The sample was secured with a rework pallet and placed onto the rework machine. The rework process involved the removal of the defective component, cleaning the component pads from the solder residue, replacing the component, and reflowing the component solder joints. The targeted reworked component, U3, was removed from the PCBA using a hot-air nozzle along with vacuum suction. Solder residues on the PCBA were removed by applying paste flux on the area and using a soldering iron and desoldering braid. The flux residue was then cleaned using a cleaning solution.

**Figure 11:**
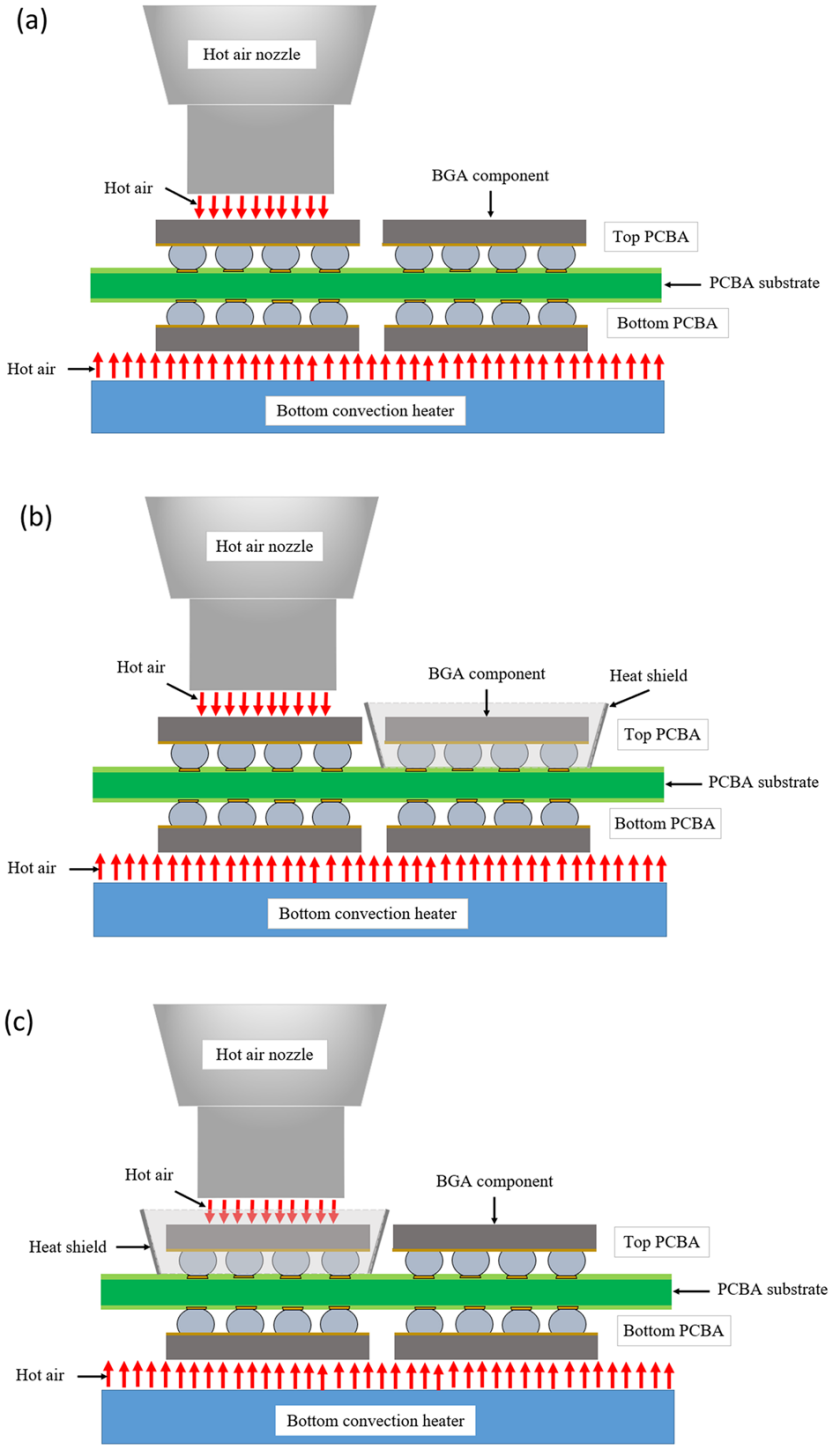
Schematic of BGA component being rework: (**a**)—without using the heat shield (sample W); (**b**)—with the heat shield on adjacent components of the rework location (sample X); (**c**)—with the heat shield on the heat source (sample Y and Z).

The reworked BGA components were assembled by applying a paste flux at the solder joints of the BGA components and then applying hot air reflow via the rework machine^28^. The hot-air source for removal and assembly came from the top nozzle and bottom convection heater of the rework machine, as shown in Figs. 11a–c. During component assembly, all solder joints must meet the melting point of lead-free alloys in the range of 217–220 °C to obtain good metallurgical bonding (IMC formation) between the solder alloys and base metals of the PCB pads^29^. For samples X, Y, and Z, both the removal and assembly of the component used heat shields to protect the adjacent components of the rework location from overheating.

### Infrared thermography

An infrared thermographic camera Fluke Ti400 was used to capture the thermal distribution on the surface of the locations of the BGA components during the rework process. Thermal imaging technology not only measures the BGA surface temperature but also provides information about heat intrusions and heterogeneity in the object's interior or subsurface^30^. This infrared thermographic camera can measure and capture the infrared radiation emitted by the BGA components during the rework process. The temperature range of this camera is from −20 to +1200 °C, which meets the rework soldering temperature range of the BGA components for the analysis.

### Dye and pull test

Dye and pull tests were held on all samples and BGA components to observe potential solder joint cracks indicators for rework and adjacent BGA components^31^. Initial optical and X-ray inspections were performed on the PCBA samples to determine signs of physical damage or stress on the reworked and adjacent components. A pull tester was used to separate the BGA components from the PCB pads. The BGA components were examined for dye indications using a Nikon Eclipse LV150NL optical microscope^32^.

### Dye penetration coverage inspection

**Figure 12:**
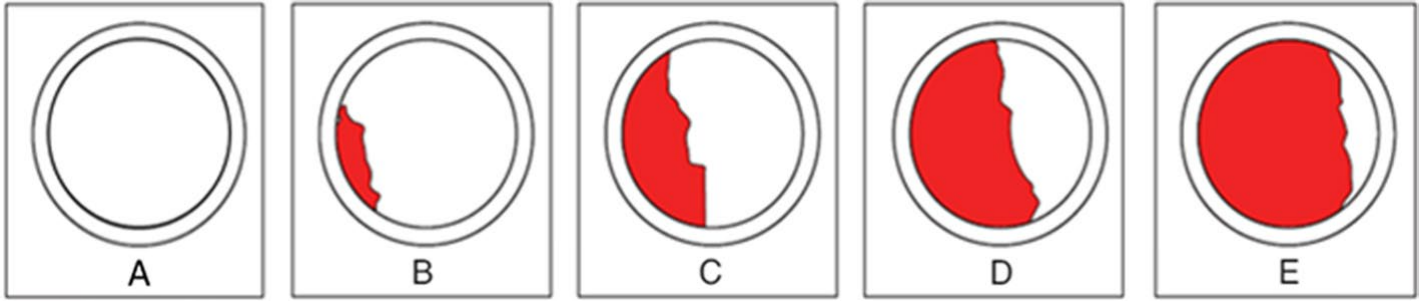
Dye penetration coverage percentage.

The BGA component solder joint was inspected for dye penetration after the BGA components were removed. The locations and percentages of dye indications were recorded and analyzed. The percentage of dye penetration coverage was calculated based on the dye coverage filling the circle quadrant, as shown in Fig. 12^33^. The percentage of dye penetration coverage values is listed in Table 3.

**Table 3:** Dye indicator and coverage percentage.

Dye indicator	Dye coverage percentage (%)
A	0
B	1 to 25
C	26 to 50
D	51 to 75
E	76 to 100

### Quantitative analysis

For quantitative analysis and calculations, the Minitab and JMP software were used complementarily. The rework-induced thermal management results were analyzed; the peak temperature variance, bar chart on quantity solder joints affected, and variability chart for dye and pull test results were generated^34^.

## Data Availability

The data that support the findings of this study are available from Western Digital®, but restrictions apply to the availability of these data, which were used under license for the current study, and so are not publicly available. Data are however available from the authors upon reasonable request and with permission of Western Digital®. All data will be available on reasonable request from corresponding author.
